# Association Between Toe Walking and Repetitive Behaviors in Children with Autism Spectrum Disorder: A Cross-Sectional Phenotypic Analysis

**DOI:** 10.3390/medicina62091764

**Published:** 2026-09-13

**Authors:** Martina Gnazzo, Giuditta Bargiacchi, Beatrice Gallai, Giulia Spoto, Gabriella Di Rosa, Valentina Baldini, Lucia Parisi, Agata Maltese, Michele Roccella, Eva Germanò, Maria Esposito, Marco Carotenuto

**Affiliations:** 1 Clinic of Child and Adolescent Neuropsychiatry, Department of Mental Health, Physical and Preventive Medicine, University of Campania “Luigi Vanvitelli”, 80131 Naples, Italygiuditta.bargiacchi@studenti.unicampania.it (G.B.); maria.esposito3@unicampania.it (M.E.); 2 IRCCS Istituto delle Scienze Neurologiche di Bologna (ISNB), 40139 Bologna, Italy; 3 The PRINCE Network (Pediatric Regulatory and Integrative Neurodevelopmental Circuits and Endophenotypes), 80131 Naples, Italygiulia.spoto27@gmail.com (G.S.); gdirosa@unime.it (G.D.R.); michele.roccella@unipa.it (M.R.); eva.germano@unime.it (E.G.); 4 Department of Surgical and Biomedical Sciences, University of Perugia, 06123 Perugia, Italy; 5 Unit of Child Neurology and Psychiatry, Department of Biomedical Sciences, Dental Sciences and Morpho-Functional Imaging, University of Messina, 98125 Messina, Italy; 6 IRCCS Centro Neurolesi Bonino-Pulejo, 98124 Messina, Italy; 7 Department of Biomedical and Neuromotor Sciences, University of Bologna, 40126 Bologna, Italy; 8 Department of Psychology, Educational Science and Human Movement, University of Palermo, 90128 Palermo, Italy; 9 Division of Child Neurology and Psychiatry, Department of the Adult and Developmental Age Human Pathology, University of Messina, 98124 Messina, Italy

**Keywords:** Autism Spectrum Disorder, toe walking, repetitive behaviors, RBS-R, sleep disturbances

## Abstract

*Background and Objectives*: Toe walking (TW) is frequently observed in children with Autism Spectrum Disorder (ASD), yet its clinical significance remains incompletely understood. While previous studies have linked TW to ASD severity and multisystem involvement, its relationship with the behavioral phenotype of repetitive behaviors has not been systematically explored. *Materials and Methods*: This study analyzed a cohort of 289 children with ASD who underwent comprehensive clinical assessment, including motor evaluation, sleep assessment using the Sleep Disturbance Scale for Children (SDSC), feeding behavior using the Brief Autism Mealtime Behavior Inventory (BAMBI), and detailed behavioral characterization through the Repetitive Behavior Scale-Revised (RBS-R) at the item level. Statistical analyses included non-parametric group comparisons, Spearman correlation analyses, and multivariable regression models. *Results*: TW was observed in 27.3% of participants. Children with TW showed significantly higher scores in stereotyped behaviors (7.71 ± 2.09 vs. 4.26 ± 2.06, r = 0.756, *p*_FDR_ < 0.001), ritualistic/sameness behaviors (11.78 ± 3.21 vs. 7.05 ± 3.03, r = 0.709, *p*_FDR_ < 0.001), and total RBS-R scores (25.52 ± 4.51 vs. 15.88 ± 4.64, r = 0.867, *p*_FDR_ < 0.001). Correlation analyses revealed a strong association between TW and stereotyped behaviors (ρ = 0.588, *p* < 0.001) and a moderate association with ritualistic/sameness behaviors (ρ = 0.549, *p* < 0.001). RBS-R total scores were also correlated with sleep disturbances (ρ = 0.532, *p* < 0.001). In the multivariate linear model, TW was associated with an adjusted mean increase of 7.77 points in the RBS-R Total Score (B = 7.771, 95% CI 6.603–8.939, *p* < 0.001, β = 0.551). In the multivariate logistic model, the combined Ritualistic/Sameness behavior domain independently predicted the presence of TW (OR = 1.549, 95% CI 1.366–1.757, *p* < 0.001). *Conclusions*: In children with ASD, TW was associated with specific domains of repetitive behavior, particularly stereotyped and ritualistic/sameness patterns. These cross-sectional findings suggest a phenotypic association between motor and behavioral features, without establishing specificity, causality, or a shared underlying mechanism. This multidimensional perspective may support future studies investigating the clinical and biological correlates of TW in ASD.

## 1. Lay Summary

Children with autism who show toe walking also tend to present more severe repetitive and ritualistic behaviors. Rather than being considered only a gait abnormality, toe walking may be a clinically relevant motor feature associated with specific patterns of repetitive behavior. These cross-sectional findings indicate a phenotypic association between these domains, but they cannot establish causality or a shared biological mechanism. Observing persistent toe walking may nevertheless provide a reason to consider a broader developmental assessment, including behavioral, sleep, feeding, and motor domains. Future studies are needed to determine whether this association is specific to autism and whether it has clinical value.

## 2. Introduction

Autism spectrum disorder (ASD) is a neurodevelopmental condition characterized by persistent difficulties in social communication and interaction together with restricted and repetitive patterns of behavior [1]. Beyond these core diagnostic features, motor abnormalities, sleep disturbances, feeding difficulties, and gastrointestinal symptoms are frequently reported in children with ASD and may contribute substantially to clinical heterogeneity and functional burden [2,3].

The prevalence of toe walking (TW) in the general population has been reported as high as 5% during early childhood, with spontaneous resolution in up to 79% of cases [4]. On the contrary, the prevalence of persistent TW in patients with neuropsychiatric conditions has been reported to be as high as 41% [4,5]. Among motor manifestations, TW is a frequent but still incompletely understood feature of ASD [6,7,8]. Previous studies have documented its prevalence and clinical associations within the autistic phenotype, ranging from 6.3% to 62.9% [9,10,11,12]. TW has also been discussed in relation to atypical sensorimotor processing, motor control, and sensory modulation, although the mechanisms underlying its occurrence in ASD remain uncertain [8,13,14,15,16].

Restricted and repetitive behaviors constitute one of the core diagnostic domains of ASD and encompass a heterogeneous range of manifestations, including motor stereotypies, ritualistic behaviors, insistence on sameness, and restricted interests [17]. These behaviors have been discussed within several neurodevelopmental frameworks as potentially related to atypical behavioral regulation and adaptation to environmental demands [17,18]. At a neurophysiological level, this behavioral inflexibility has been linked to a reduced capacity to dynamically modulate and shift brain states across large-scale neurocognitive networks [19]. Repetitive motor patterns have also been hypothesized to have potential attentional or regulatory functions, although their specific significance has yet to be established [20]. Neuroimaging studies have further reported associations between specific patterns of cortical thickness and the severity of restricted and repetitive behaviors in ASD [21].

Sleep disturbances are among the most prevalent comorbidities in ASD and have important implications for daytime functioning and clinical management [22,23]. Several studies have also reported associations between sleep disturbances and greater sensory-processing difficulties, repetitive behaviors, compulsive behaviors, and self-injurious behaviors [24,25,26,27]. Although these associations suggest potential interactions among these clinical domains, the mechanisms underlying these relationships remain incompletely understood.

Within this perspective, a dimensional and integrative approach becomes essential. Rather than examining individual domains in isolation, the simultaneous assessment of motor patterns, repetitive behaviors, and sleep disturbances may provide a more accurate representation of the clinical phenotype.

The present study adopts this approach by examining TW not merely as an isolated gait characteristic, but as a motor feature potentially associated with a broader pattern of repetitive-behavior characteristics within ASD.

Accordingly, this cross-sectional secondary analysis was designed to investigate whether TW is selectively associated with specific domains of restricted and repetitive behaviors after accounting for sleep disturbances, feeding selectivity, autism severity, age, and sex. We hypothesized that TW would be associated with selected behavioral domains rather than uniformly with the overall severity of repetitive behaviors.

Although this study uses the same multicenter cohort described by Costanza et al. [9], its scientific objectives differ substantially. The previous publication focused on the prevalence of TW and its general clinical correlates in children with ASD. In contrast, the present study represents a hypothesis-driven secondary phenotypic analysis designed to investigate whether TW is selectively associated with specific domains of restricted and repetitive behaviors using multivariable statistical models adjusted for relevant clinical covariates. Thus, the present manuscript addresses a distinct research question and reports analyses that were not included in the previous publication.

## 3. Methods

### 3.1. Ethical Statement

The study was approved by the Institutional Review Board of the University of Campania “Luigi Vanvitelli,” which served as the coordinating and leading center for study oversight, centralized data management, and statistical analysis (protocol no. 217; approval date: 4 August 2020). This study is a secondary, hypothesis-driven analysis of a prospectively collected multicenter observational cohort. The parent cohort was subsequently registered on ClinicalTrials.gov (NCT07122388); however, this registration occurred after participant enrollment had already begun. Accordingly, the present secondary hypothesis-driven analysis should not be considered prospectively registered.

### 3.2. Study Design and Population

This study is a secondary hypothesis-driven analysis of a previously collected multicenter observational dataset including children diagnosed with ASD. The original cohort comprised pediatric patients recruited between September 2020 and June 2023 across four Italian academic clinical centers (University of Palermo, University of Perugia, University Kore of Enna, and UniCamillus-Saint Camillus International University of Health Sciences of Rome), with the University of Campania ‘Luigi Vanvitelli’ serving as the coordinating center. At each site, experienced, licensed child neuropsychiatrists with specific expertise in child neurology established ASD diagnoses according to DSM-5 criteria or DSM-5-TR criteria and confirmed them through a standardized clinical evaluation, ensuring diagnostic consistency across all recruiting centers. To ensure diagnostic and severity consistency across the entire study population, all cases initially evaluated under the DSM-5 were subsequently reviewed and reconfirmed in accordance with the updated DSM-5-TR criteria once published. Severity levels were classified into Level 1, Level 2, and Level 3, reflecting increasing degrees of support required. The child neuropsychiatrist responsible for each patient assigned DSM-5-TR severity levels at the time of the diagnostic assessment, based on the level of support required across the two core diagnostic domains and integrating clinical observation, developmental history, caregiver interview, and standardized assessment findings. All participants were completely drug-naive at the time of the evaluation, with no history of receiving antipsychotics, psychostimulants, or other medications known to affect motor function, behaviour, or sleep patterns.

Only participants with a confirmed diagnosis of ASD and complete data on motor, sleep, and behavioural measures were included in the present analysis. Typically developing controls were excluded from the current study, as the focus was on phenotypic characterization within the ASD population.

**Figure 1:**
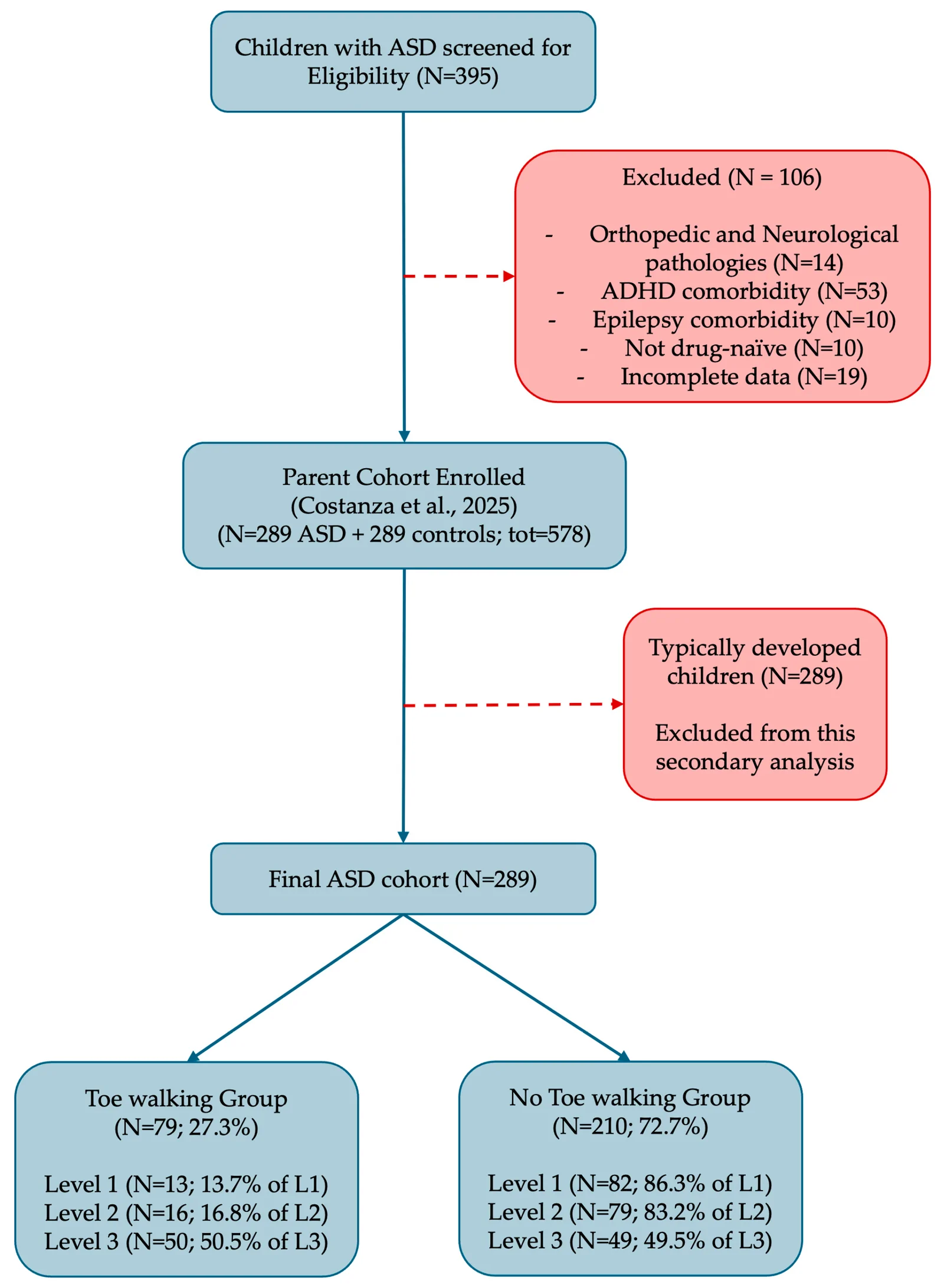
Participant selection, exclusion, and phenotypic stratification flowchart. The flowchart details the multi-step screening and selection process for the study cohort. From an initial pool of 395 children clinically evaluated for ASD across four academic centers, 106 patients were excluded during the screening phase due to orthopedic and neurological pathologies, ADHD and epilepsy comorbidity, current psychotropic treatment, or incomplete datasets. The remaining parent cohort of 289 children with ASD and 289 matched typically developing controls (TDC) was originally enrolled as described by Costanza et al. [9]. For this secondary analysis, we excluded the TDC group to focus strictly on behavioural–motor phenotypic characterization within the ASD population. The final analyzed ASD cohort is stratified by TW status, identifying 79 children with TW (27.3%) and 210 without TW (72.7%). The sample has been further classified according to DSM-5-TR severity levels (Level 1, Level 2, and Level 3), highlighting the significant clinical gradient of TW prevalence across severity subgroups.

The detailed participant selection process—including initial screening, exclusion of clinical confounders, and the selection of the final 289 complete-case ASD participants analyzed in this study—is illustrated in the Participant Flow Diagram (Figure 1).

All participating centers applied a common clinical assessment protocol using the same standardized instruments and validated Italian versions. Experienced clinicians performed the clinical assessments, with established expertise in administering and interpreting these instruments.

Although the study population overlaps with that reported by Costanza et al. [9], the objectives, outcome measures, and statistical analyses presented herein are novel and were not reported in the previous publication.

### 3.3. Clinical and Behavioral Assessment

#### 3.3.1. Toe Walking (TW)

TW was assessed by direct clinical observation during spontaneous barefoot walking as part of the routine neurological and neurodevelopmental examination performed by experienced child neuropsychiatrists. Gait was observed from the child’s spontaneous entrance into the examination room and throughout the clinical evaluation, allowing the assessment of naturally occurring walking behaviour under routine clinical conditions. Classification was based on a consistent pattern of spontaneous TW observed during the examination rather than on an isolated or transient episode. The assessment followed the same clinical criteria and operational approach previously described by Costanza et al. [9]. The evaluation was intentionally conducted under routine clinical conditions rather than according to a standardized gait-analysis protocol. This approach was considered particularly appropriate because a substantial proportion of participants had moderate-to-severe ASD, in whom compliance with standardized motor instructions is frequently limited and may itself modify the natural expression of gait behaviour. Consequently, we aimed to identify a clinically observable motor phenotype rather than quantify gait biomechanics. All participants underwent a comprehensive neurological and musculoskeletal examination. In addition, we referred all children for specialist physiatric and orthopedic evaluation when cooperation with the examination was possible and when clinically indicated, particularly in cases of diagnostic uncertainty or when further specialist assessment was considered necessary, to confirm the clinical characterization of TW and to investigate and exclude alternative neurological, orthopedic, or musculoskeletal causes of gait abnormalities. Accordingly, we excluded children with clinical evidence of cerebral palsy, neuromuscular disorders, peripheral neuropathies, clinically relevant orthopedic deformities, fixed Achilles’ tendon contracture, spasticity, abnormal neurological findings, or other medical conditions affecting gait. TW was ultimately classified as present or absent according to this multidisciplinary clinical assessment. Children who exhibited only occasional or isolated TW during the examination were not classified as having TW.

#### 3.3.2. Sleep Habits Assessment

Sleep disturbances were evaluated using the Sleep Disturbance Scale for Children (SDSC), a validated parent-reported questionnaire assessing multiple domains of sleep. The questionnaire is completed by a parent or caregiver in approximately 10–15 min using a paper-and-pencil format. Each item is scored on a 5-point Likert scale ranging from 1 (“never”) to 5 (“always/daily”), with higher scores reflecting greater sleep disturbance severity. Scores are calculated for each of the six sleep-disorder domains (i.e., disorders of initiating and maintaining sleep, sleep breathing disorders, disorders of arousal/nightmares, sleep–wake transition disorders, disorders of excessive somnolence, and sleep hyperhidrosis), and an overall total score is subsequently derived. The total score was used as an index of global sleep dysregulation, with higher values indicating greater severity of sleep disturbances [28]. The Italian version of the SDSC has good internal consistency (Cronbach’s α = 0.79 in the Control group and 0.71 in the Sleep Disorders group) and satisfactory test–retest and inter-rater reliability [28]. In our study sample, the internal consistency of the SDSC Total Score, computed by treating the six subscales as component scores, was acceptable (Cronbach’s α = 0.69).

#### 3.3.3. Feeding Behavior

Feeding and mealtime behaviors were assessed using the Brief Autism Mealtime Behavior Inventory (BAMBI). Total scores were used as a measure of feeding rigidity and selectivity, with higher values reflecting greater behavioral difficulties during mealtime. The BAMBI was developed to evaluate mealtime behaviors in children aged 3–11 years, regardless of ASD diagnosis. It consists of 18 items rated on a 5-point Likert scale ranging from 1 (“never”) to 5 (“always”), with four items reverse-scored. Higher total scores indicate greater severity of problematic mealtime behaviors. Factor analysis identified three domains: Limited Variety, Food Refusal, and Features of Autism. Although item-level data were not retained in this anonymized secondary database to directly compute current-sample internal consistency, the Italian version demonstrated good internal consistency (Cronbach’s α = 0.86 in children with typical development and 0.71 in children with ASD) and satisfactory test–retest and inter-rater reliability [29].

#### 3.3.4. Assessment of Repetitive Behaviors

Repetitive behaviors were assessed using the Italian version of the Repetitive Behavior Scale-Revised (RBS-R) [30], a parent-reported instrument designed to capture the full spectrum of restricted and repetitive behaviors in individuals with ASD. The RBS-R consists of 43 items, each rated on a 4-point Likert scale ranging from 0 (behavior does not occur) to 3 (behavior occurs frequently and is a severe problem). Items are grouped into six subdomains: stereotyped behavior, self-injurious behavior, compulsive behavior, ritualistic behavior, sameness behavior, and restricted interests.

In the present study, RBS-R data were modeled at the item level, with all item scores retained as integer values. Subscale scores were computed as the sum of item scores within each domain, and a total score was obtained by summing all subscales. While the RBS-R items were originally conceptually grouped into six subscales, for the purpose of this study we adopted the five-factor solution validated by Lam and Aman [31]. Specifically, the original “Ritualistic” and “Sameness” subscales were combined into a single “Ritualistic/Sameness Behavior” domain. This approach is based on psychometric evidence suggesting that these behaviors share an underlying construct related to the need for invariance in both activities and the environment, and that a five-factor structure is more statistically sound and stable for clinical samples of children with ASD [31].

In addition, the Global Rating Score (range: 1–100) was included to reflect the parental perception of the overall functional interference of repetitive behaviors in daily life, with higher values indicating greater perceived impairment [30]. In the present sample, the observed Cronbach’s alpha was 0.620 for the RBS-R Total Score, 0.488 for Stereotyped behaviors, and 0.502 for Sameness behaviors. The remaining individual subscales (Self-Injurious, Compulsive, Ritualistic, and Restricted Interests) exhibited very low internal consistency (ranging from 0.018 to 0.176). These low coefficients are consistent with previous psychometric literature on very young, preschool-aged pediatric ASD samples, where repetitive behaviors are highly situational, heterogeneous, and emerging, as previously highlighted in the Italian validation of the scale [30] and in the independent psychometric validation of the instrument [31]. To address this psychometric constraint and ensure statistical stability, multivariable regression models were restricted to the RBS-R Total Score and the combined Ritualistic/Sameness subdomain.

For each participant, the SDSC, BAMBI, and RBS-R were completed by the same primary caregiver. Questionnaire administration and clinical examination were performed as part of the routine multidisciplinary clinical assessment on the same day as part of the same assessment session. The clinical assessment of TW was performed independently through direct observation and was not derived from questionnaire responses.

### 3.4. Data Integration and Phenotypic Framework

To explore the multidimensional clinical presentation of ASD, motor, repetitive-behavior, sleep, and feeding variables were examined together to characterize clinical co-occurrence across multiple domains. TW was considered as a motor phenotype, RBS-R scores as measures of repetitive behavioral characteristics, SDSC scores as measures of sleep disturbances, and BAMBI scores as an index of feeding-related behavioral features. These clinical domains were discussed within the broader conceptual framework of *The PRINCE Network* (Pediatric Regulatory and Integrative Neurodevelopmental Circuits and Endophenotypes), which provides a theoretical perspective for considering the co-occurrence of motor, behavioral, sleep, and feeding-related features in neurodevelopmental disorders. However, no direct measure of a shared regulatory system was included in the present study, and the present analyses were not designed to test a regulatory-systems model.

### 3.5. Statistical Analysis

All statistical analyses were performed using IBM SPSS Statistics version 29.0 (IBM Corp., Armonk, NY, USA).

Descriptive statistics were calculated for all variables, including means, standard deviations, medians, and interquartile ranges where appropriate. Normality of distribution was assessed using the Shapiro–Wilk test. Given that several variables showed non-normal distribution, non-parametric analyses were applied.

Group differences between children with and without TW were evaluated using the Mann–Whitney *U* test for continuous variables and the chi-square test for categorical variables. For the non-parametric group comparisons (Mann–Whitney *U*), effect sizes were calculated using the rank-biserial correlation coefficient (r). To ensure intuitive readability for the reader, these coefficients are reported in absolute terms (positive values), where a positive value consistently represents higher behavioral or clinical scores in the TW group relative to the non-TW group.

Associations between continuous and ordinal variables —including RBS-R subscales, sleep disturbances (SDSC), feeding behavior (BAMBI), and ASD severity levels— were examined using Spearman’s rank correlation coefficient (ρ) and exact *p*-values. Because TW was dichotomous, Spearman’s rho was retained for the exploratory correlation matrix to provide a uniform association measure across variables, whereas for direct comparisons between the TW groups, rank-biserial correlations were reported as non-parametric effect sizes. Exploratory correlations involving ASD severity were based on ordinal coding of the DSM-5-TR levels. To further explore the relationship between repetitive behaviors and clinical variables, a multiple linear regression model was performed with RBS-R total score as the dependent variable. Independent variables included TW, SDSC total score, BAMBI score, ASD severity level (dummy-coded using Level 1 as the reference category), age, and sex (dummy-coded: Male = 1, Female = 0). Linear model assumptions were verified post hoc (Breusch–Pagan, Shapiro–Wilk on residuals, Cook’s distance, and Variance Inflation Factors < 5.0).

Additionally, a binary logistic regression analysis was conducted to identify variables independently associated with TW. Independent variables included RBS-R Ritualistic/Sameness score, SDSC total score, BAMBI score, chronological age, sex (dummy-coded: Male = 1, Female = 0), and dummy-coded ASD severity levels (using Level 1 as the reference category).

Significance was set at *p* < 0.05, with Benjamini–Hochberg False Discovery Rate (FDR) correction applied to all univariate baseline and item-level comparisons. FDR correction was applied within three independent families of hypotheses: (1) baseline clinical and demographic characteristics (Family 1; comparisons for general clinical indices, overall sleep, and overall feeding scores); (2) RBS-R subscale and total score comparisons (Family 2; comparisons for repetitive behavior domains); and (3) exploratory item-level comparisons across all 43 individual RBS-R behaviors (Family 3). For each family of hypotheses, both raw *p*-values (*p*) and FDR-adjusted *p*-values (*p*_FDR_) are reported to ensure statistical transparency. The bivariate correlation matrix was analyzed separately for descriptive purposes and was not subjected to FDR correction.

## 4. Results

### 4.1. Sample Characteristics

The final clinical sample comprised 289 children with ASD (mean age 5.92 ± 0.78 years, median 5.90, IQR [5.40–6.50]; range: 4.8–7.0 years). Of the total sample, 73 participants (25.3%) were female and 216 (74.7%) were male. Within this cohort, 79 children (27.3%) presented with a clinically observed TW, whereas 210 children (72.7%) did not (non-TW). The clinical and demographic characteristics of the TW and non-TW subgroups are summarized in Table 1.

No significant differences were detected in the TW and non-TW groups with respect to age (TW: 5.94 ± 0.77 years vs. non-TW: 5.91 ± 0.78 years; Mann–Whitney *U* = 8443.5, *p*_FDR_ = 0.811, r = −0.018). Regarding sex distribution, the proportion of female children was significantly higher in the TW group than in the non-TW group (43.0% vs. 18.6%). When stratifying TW prevalence by sex in the entire cohort, the prevalence of TW was significantly higher in females than in males: TW was present in 46.6% of female patients (34/73) compared to 20.8% of male patients (45/216) (χ^2^ = 16.93, *p*_FDR_ < 0.001, Cramer’s *V* = 0.242).

Importantly, TW was strongly associated with overall autism severity; children in the TW group exhibited significantly higher ADOS-2 total scores (median 18.00, IQR [13.00–20.00] vs. median 13.00, IQR [11.00–15.00]; *U* = 11,655.0, *p*_FDR_ < 0.001, r = 0.405) and ADI-R scores (median 30.00, IQR [27.00–32.00] vs. median 27.00, IQR [20.00–29.00]; *U* = 11,015.5, *p*_FDR_ < 0.001, r = 0.328). Regarding autism severity, the proportion of children classified as Level 3 was significantly higher in the TW group than in the non-TW group (63.3% vs. 23.3%). When stratifying TW prevalence by DSM-5-TR severity levels across the entire cohort, a significant non-random distribution was observed: the prevalence of TW was 13.7% in Level 1 (13/95), 16.8% in Level 2 (16/95), and rose sharply to 50.5% in Level 3 (50/99) (χ^2^ = 40.94, *p*_FDR_ < 0.001, Cramer’s *V* = 0.376).

Regarding somatic and regulatory co-occurrences, children with TW showed significantly higher caregiver-reported sleep disturbances (SDSC Total score: 74.70 ± 8.12, median 76.00, IQR [69.50–80.50] vs. 68.28 ± 7.97, median 68.00, IQR [62.00–73.00]; *U* = 11,830.5, *p*_FDR_ < 0.001, r = 0.426). This sleep dysregulation was consistently observed across almost all SDSC subscales, including Sleep-Related Breathing Disorders (SRBD; *p*_FDR_ < 0.001), Disorders of Arousal (DA; *p*_FDR_ = 0.005), Sleep–Wake Transition Disorders (DWST; *p*_FDR_ < 0.001), Disorders of Excessive Somnolence (DOES; *p*_FDR_ < 0.001), and Sleep Hyperhidrosis (SHY; *p*_FDR_ < 0.001). Similarly, children in the TW group presented with more severe feeding issues (BAMBI Score: median 80.00, IQR [60.00–86.00] vs. median 62.00, IQR [58.00–83.75]; *U* = 10,074.0, *p*_FDR_ = 0.005, r = 0.214) and a significantly higher rate of functional constipation (64.6% vs. 35.7%; χ^2^ = 18.27, *p*_FDR_ < 0.001, ϕ = 0.251).

### 4.2. Distribution of Repetitive Behaviors

Across the entire cohort of children with ASD (*N* = 289), restricted and repetitive behaviors assessed through the RBS-R showed a wide dimensional distribution.

The overall cohort presented a mean RBS-R Total Score of 18.51 ± 6.30 (median: 18.00, IQR: [14.00–22.00], while the Global Rating Score showed a mean of 40.33 ± 16.54 (median: 40.00, IQR: [29.00–52.00]). When examining individual RBS-R subdomains, the most prominent and highly endorsed behavioral patterns in the cohort belonged to the Ritualistic/Sameness subdomain, with a mean score of 8.34 ± 3.73 (median: 8.00, IQR: [6.00–11.00]), and the Stereotyped Behavior subdomain, with a mean score of 5.20 ± 2.57 (median: 5.00, IQR: [3.00–7.00]). In contrast, lower mean scores and narrower distributions were observed for Compulsive Behavior (mean: 2.17 ± 1.53, median: 2.00, IQR: [1.00–3.00]), Restricted Interests (mean: 2.15 ± 1.34, median: 2.00, IQR: [1.00–3.00]), and Self-Injurious Behavior (mean: 0.65 ± 0.85, median: 0.00, IQR: [0.00–1.00]). These subdomain distributions appeared positively skewed across the cohort, driven primarily by strong floor effects in the self-injurious, compulsive, and restricted interest domains, which is characteristic of emerging repetitive phenotypes in preschool-aged children. Overall, these findings indicate variability in the dimensional expression of core repetitive behaviors across the recruited pediatric sample, with the highest mean scores observed for Ritualistic/Sameness and Stereotyped Behavior, whereas Compulsive, Restricted Interests, and Self-Injurious Behavior showed lower mean scores.

### 4.3. Association Between Toe Walking and Repetitive Behaviors

**Figure 2:**
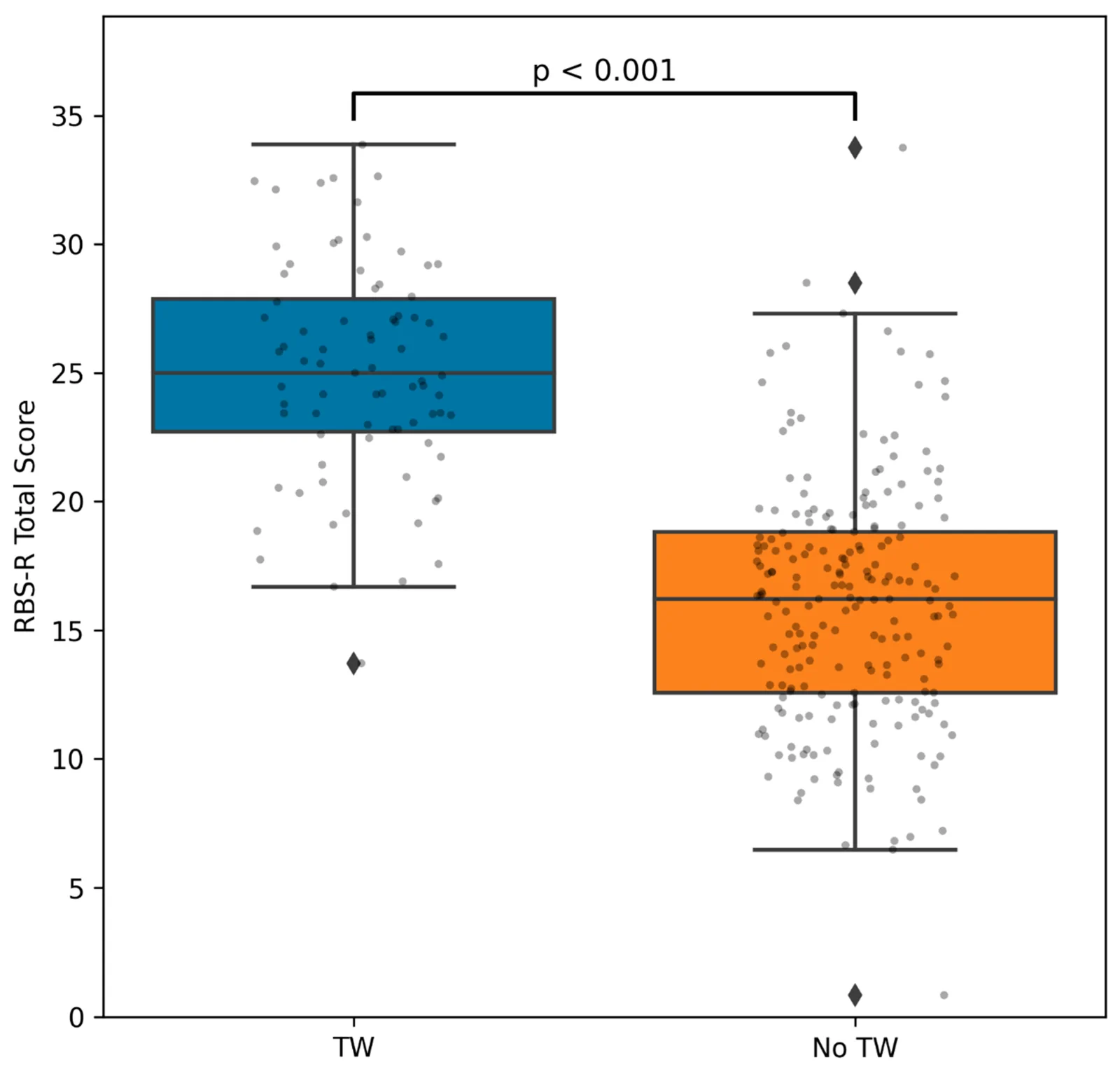
Distribution of RBS-R total scores in children with (*N* = 79) and without (*N* = 210) TW. Box plots display the median (horizontal line), IQRs (box), and whiskers extending to the furthest data points within 1.5 times the IQR from the hinges (Tukey convention). Individual participant values are overlaid as jittered dots to illustrate sample density. Repetitive behavior severity is significantly higher in the TW group than in the non-TW group (median 26.0 [IQR: 22.0–28.0] vs. 16.0 [IQR: 13.0–19.0]; Mann–Whitney *U* = 15,488.0, *p* < 0.001, rank-biserial correlation r = 0.867, *p*_FDR_ < 0.001). Legend: IQR = interquartile range; RBS-R = Repetitive Behavior Scale-Revised; TW = toe walking.

In unadjusted group comparisons, children in the TW group showed significantly higher scores across all RBS-R subscales and in the overall total score compared with the non-TW group. The RBS-R Total Score was higher in the TW group, with a mean of 25.52 ± 4.51, a median of 26.00, and an IQR of [22.00–28.00], compared to the non-TW group, which presented a mean of 15.88 ± 4.64, a median of 16.00, and an IQR of [13.00–19.00]. This difference represented a significant clinical effect (Mann–Whitney *U* = 15,488.0, *p*_FDR_ < 0.001, rank-biserial correlation r = 0.867) (Figure 2).

Caregiver-perceived behavioral burden, captured by the Global Rating Score, was also significantly elevated in children with TW, exhibiting a mean of 49.66 ± 16.20, a median of 51.00, and an IQR of [37.00–61.50], compared to the non-TW group, which showed a mean of 36.82 ± 15.29, a median of 37.00, and an IQR of [27.00–48.50] (*U* = 11,915.5, *p* < 0.001, r = 0.436).

**Figure 3:**
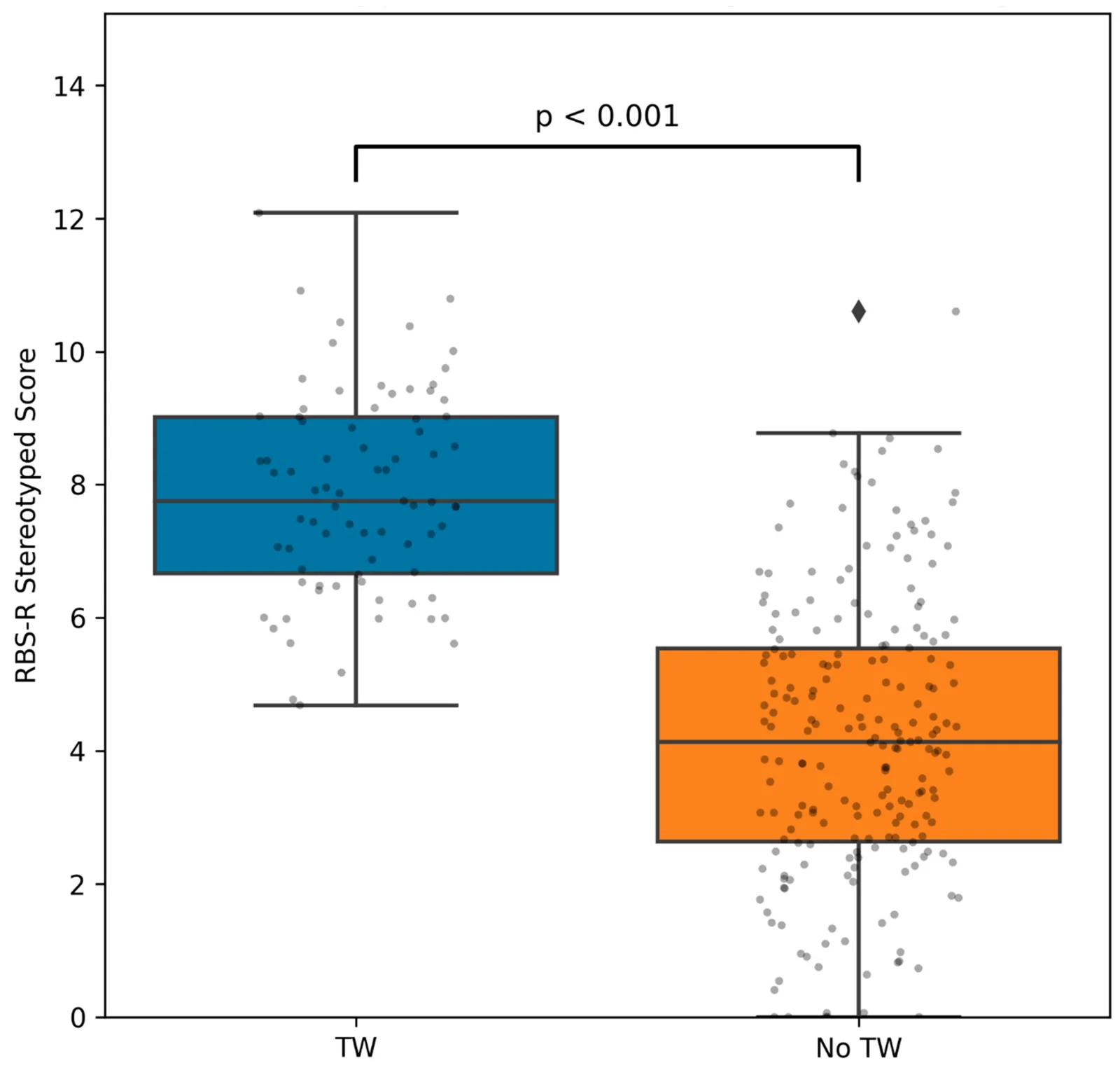
Distribution of RBS-R Stereotyped Behavior subscale scores stratified by TW status (TW, *N* = 79; non-TW, *N* = 210). Box plots represent medians, IQRs, and whiskers extending to the furthest data points within 1.5 times the IQR from the hinges (Tukey convention), with individual participant data points overlaid as jittered dots. Stereotyped behaviors are significantly more severe in children presenting with TW compared to those without (median 8.0 [IQR: 6.0–9.0] vs. 4.0 [IQR: 3.0–6.0]; Mann–Whitney *U* = 14,563.5, *p* < 0.001, rank-biserial correlation r = 0.756, *p*_FDR_ < 0.001).

At the subdomain level, the largest differences between the two groups were observed in stereotyped behaviors and the combined ritualistic–sameness subdomain. Stereotyped behavior scores were elevated in the TW group (mean: 7.71 ± 2.09, median: 8.00, IQR: [6.00–9.00]) compared to the non-TW group (mean: 4.26 ± 2.06, median: 4.00, IQR: [3.00–6.00]; *U* = 14,563.5, *p*_FDR_ < 0.001, r = 0.756) (Figure 3).

Similarly, the combined ritualistic–sameness score was higher in the TW group, with a mean of 11.78 ± 3.21, a median of 12.00, and an IQR of [9.50–14.00], than in the non-TW group, which presented a mean of 7.05 ± 3.03, a median of 7.00, and an IQR of [5.00–9.00] (*U* = 14,178.5, *p*_FDR_ < 0.001, r = 0.709). For the individual subscales composing this combined domain, both showed significant elevations in TW children, with ritualistic behaviors scoring higher in the TW group (mean: 4.08 ± 1.65, median: 4.00, IQR: [3.00–5.00] vs. mean: 3.02 ± 1.60, median: 3.00, IQR: [2.00–4.00]; *U* = 11,333.0, *p*_FDR_ < 0.001, r = 0.366) and sameness behaviors exhibiting a large difference between groups (mean: 7.71 ± 2.35, median: 8.00, IQR: [6.00–9.00] vs. mean: 4.03 ± 2.49, median: 4.00, IQR: [2.00–5.00]; *U* = 14,265.0, *p*_FDR_ < 0.001, r = 0.720). Significant differences, with smaller effect sizes, were also confirmed for all other subscales. Specifically, compulsive behaviors were significantly higher in the TW group (median: 3.00, IQR: [2.00–4.00]) compared to the non-TW group (median: 2.00, IQR: [1.00–3.00]; *U* = 10,200.5, *p*_FDR_ = 0.003, r = 0.230), and restricted interests showed a similar pattern with a median of 3.00, IQR [2.00–3.00] in the TW group versus 2.00, IQR [1.00–3.00] in the non-TW group (*U* = 10,568.5, *p*_FDR_ < 0.001, r = 0.274). Finally, self-injurious behaviors, while generally exhibiting low rates across the sample, were also significantly more pronounced in TW children, with a median of 1.00, IQR [0.00–1.00] compared to a median of 0.00, IQR [0.00–1.00] in the non-TW group (*U* = 9896.0, *p*_FDR_ = 0.005, r = 0.193).

These unadjusted group differences were subsequently evaluated using multivariable models to determine whether the observed associations remained after adjustment for relevant clinical covariates. An exploratory, FDR-corrected item-level analysis of all 43 RBS-R items was conducted to further dissect the specific behavioral topography associated with TW. Out of 43 items, 21 remained significantly different between the TW and non-TW groups after stringent multiple-testing correction (all *p*_FDR_ < 0.05; reported in full in Supplementary Table S1). The items showing the strongest associations with the presence of TW belonged primarily to the stereotyped and ritualistic/sameness domains, which included body movements (r = 0.397, *p*_FDR_ < 0.001), finger movements (r = 0.416, *p*_FDR_ < 0.001), locomotion (r = 0.467, *p*_FDR_ < 0.001), videotapes (r = 0.335, *p*_FDR_ < 0.001), and insistence on routine (r = 0.418, *p*_FDR_ < 0.001).

### 4.4. Relationship Between Repetitive Behaviors and Sleep Disturbances

In investigating the clinical co-occurrence between sleep dysregulation and repetitive behavior severity, we observed a moderate but significant positive correlation between the overall caregiver-reported scores. Specifically, the RBS-R Total Score and the SDSC total score exhibited a significant positive correlation (ρ = 0.532, *p* < 0.001), indicating that higher levels of repetitive behavior were associated with greater sleep disturbance.

When examining individual subscales, this association was particularly characterized by the higher-order insistence on sameness. The combined ritualistic/sameness behavior subdomain showed a moderate and significant positive correlation with the SDSC total score (ρ = 0.463, *p* < 0.001), whereas stereotyped behaviors showed a weaker, though still statistically consistent, relationship with overall sleep quality. Furthermore, the Global Rating Score was also significantly correlated with the SDSC total score (ρ = 0.493, *p* < 0.001).

### 4.5. Correlation Analysis

To further dissect the dimensional relationships across motor, behavioral, and clinical severity domains in the ASD cohort, a systematic bivariate correlation analysis was performed. As summarized in Table 2, clinically observed TW exhibited selective and domain-specific relationships with specific repetitive behavior subscales.

Specifically, the presence of TW showed a moderate-to-strong association with stereotyped behaviors (ρ = 0.588, *p* < 0.001) and a moderate association with combined ritualistic/sameness behaviors (ρ = 0.549, *p* < 0.001), while showing a strong correlation with the overall RBS-R Total Score (ρ = 0.670, *p* < 0.001).

In contrast, the association between the core behavioral construct (RBS-R Total Score) and other clinical parameters showed that overall repetitive behavior severity was significantly and positively associated with global autism severity as indexed by the DSM-5-TR ASD Level (ρ = 0.535, *p* < 0.001), but showed no significant relationship with chronological age (ρ = −0.058, *p* = 0.325), suggesting that the dimensional expression of repetitive phenotypes remains relatively stable across the narrow preschool age range investigated in this sample.

**Figure 4:**
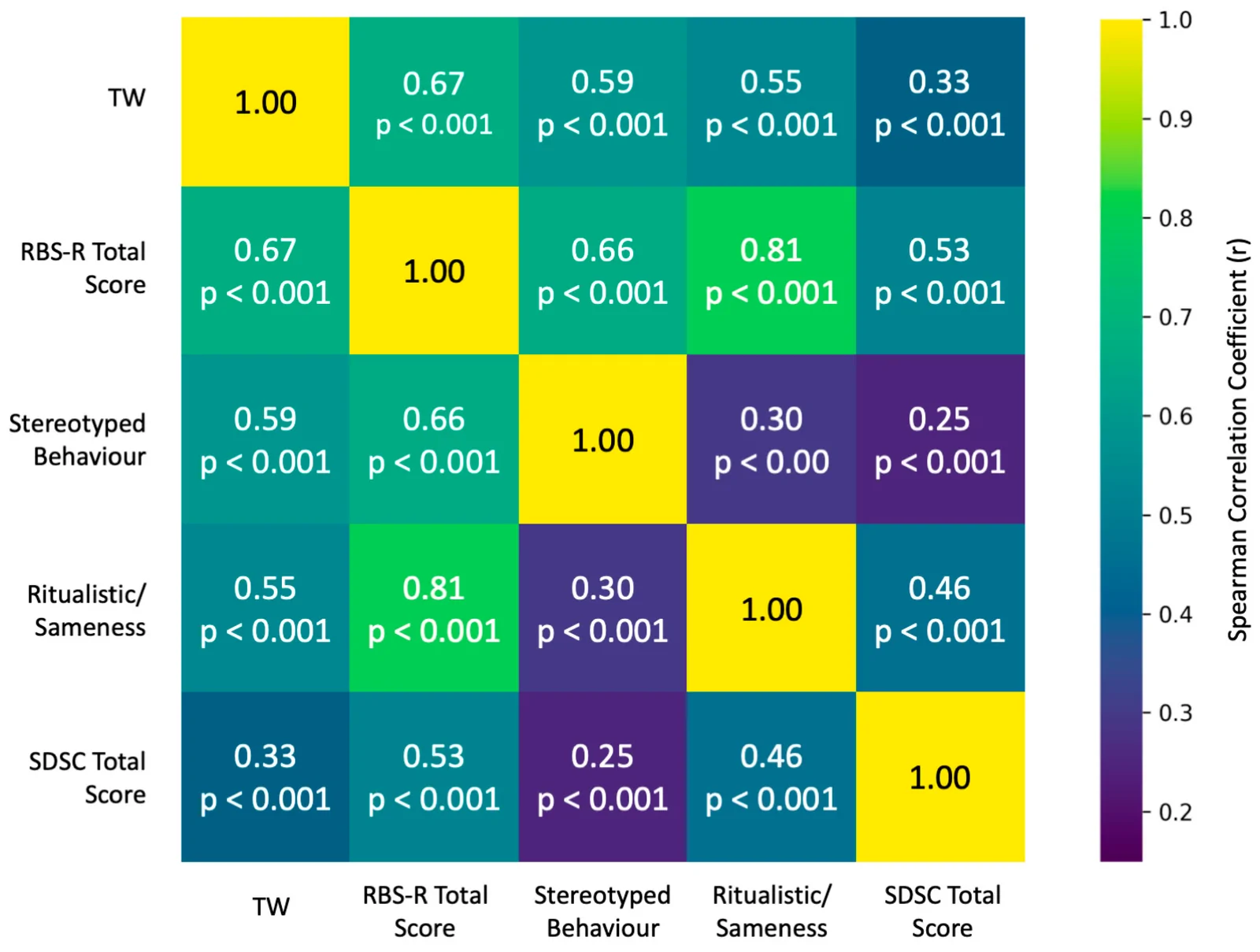
Spearman rank correlation matrix showing the dimensional relationships between clinical motor presentation (clinically observed TW status), repetitive behavior domains (RBS-R Total, Stereotyped, and combined Ritualistic/Sameness scores), and sleep disturbances (SDSC Total score) in the ASD cohort (*N* = 289). TW was coded as 0 = absent and 1 = present; therefore, the corresponding Spearman coefficients represent associations involving a dichotomous variable. Cell values display the Spearman correlation coefficient. *p*-values were calculated using two-sided Spearman rank correlation tests; all bivariate associations were statistically significant (*p* < 0.001). Statistical significance across all bivariate associations was significant (*p* < 0.001). Legend: ASD = Autism Spectrum Disorder; RBS-R = Repetitive Behavior Scale-Revised; SDSC = Sleep Disturbance Scale for Children; TW = Toe Walking.

Bivariate associations across variables are presented in the exploratory correlation matrix in Figure 4, while non-parametric rank-biserial correlation coefficients (r) are reported for direct comparisons between the TW and non-TW groups. The correlation matrix shows moderate-to-strong positive correlations between clinically observed TW, repetitive behavior domains, and sleep disturbances across the cohort.

### 4.6. Multiple Linear Regression: Variables Associated with Repetitive Behaviors

To determine whether TW is independently associated with overall repetitive behavior severity, we constructed a multivariable linear regression model with the RBS-R Total Score as the continuous dependent variable. Covariates entered into the model included clinically observed TW, SDSC total score, BAMBI score, age, sex, and ASD severity levels treated as dummy-coded categorical factors.

The overall multivariable model was significant, with an R^2^ of 0.606, indicating that the model accounted for 60.6% of the variance in repetitive behaviors. Clinically observed TW emerged as the strongest independently associated factor with the RBS-R Total Score. Specifically, after adjusting for age, sex, global clinical severity, sleep disturbances, and feeding selectivity, the presence of TW was associated with an average increase of approximately 7.77 points on the RBS-R Total Score. Caregiver-reported feeding selectivity, assessed through the BAMBI Score, and clinical autism severity (both Level 2 and Level 3) also emerged as significant independently associated variables. Conversely, sleep disturbances, chronological age, and sex did not reach independent statistical significance in this multivariable context.

To ensure the mathematical validity and robustness of these findings, a rigorous series of post hoc diagnostic checks was performed on the regression model. Standardized residuals were compatible with a normal distribution, as confirmed by the Shapiro–Wilk test (*W* = 0.991, *p* = 0.083). The Breusch–Pagan test showed no evidence of heteroscedasticity (*p* = 0.177), and the Durbin–Watson statistic of 1.940 ruled out any relevant autocorrelation in the residuals. Influential outlier analysis using Cook’s distance confirmed that no single observation exerted an undue influence on the estimated coefficients, with a maximum Cook’s distance of 0.036, which is well below the standard conservative threshold of 1.0. Multicollinearity was formally assessed using Variance Inflation Factors (VIFs) for all covariates. Most independent variables exhibited VIF values well below the conservative threshold of 5.0 (ranging from 1.005 to 3.821), while the dummy-coded variable representing ASD Level 3 severity showed a VIF of 5.400 (Tolerance = 0.185). Although slightly exceeding the conservative threshold of 5.0, this value remained well below the commonly used threshold of 10.0 for severe multicollinearity. Importantly, the primary exposure of interest, TW, showed a very low VIF (1.262; Tolerance = 0.792), indicating minimal collinearity with the other covariates.

The complete model coefficients, confidence intervals, and collinearity diagnostics are summarized in Table 3.

### 4.7. Multivariable Logistic Regression: Variables Associated with Toe Walking

To evaluate the specific behavioral phenotype associated with the presence of TW, a binary logistic regression model was constructed with TW as the binary outcome. Covariates included the combined RBS-R Ritualistic/Sameness score, SDSC total score, BAMBI score, age, sex, and ASD severity levels. The multivariable model was restricted to the combined Ritualistic/Sameness subdomain because this was the primary behavioral construct hypothesized to be most closely related to the observed TW phenotype [32]. Other RBS-R subdomains were not included in the multivariable analysis because there was insufficient established literature or a specific clinical rationale supporting their independent motor or postural association with TW. This approach also reduced the number of predictors included in the model, thereby limiting the risk of overfitting and promoting greater statistical stability.

Before conducting multivariable modeling, multicollinearity among the six standard RBS-R subscales was formally assessed. Bivariate Spearman rank correlations among the subscales in our sample were modest (ranging from 0.03 to 0.31), and VIFs were uniformly low (range: 1.033 to 1.169, tolerance ≥ 0.855), indicating minimal collinearity among these domains and no evidence of problematic multicollinearity. For the final model, VIF values were 1.338 for the combined Ritualistic/Sameness score, 3.873 for the SDSC Total score, 1.387 for the BAMBI score, 1.004 for age, 1.010 for sex, 1.972 for Level 2 severity, and 5.375 for the Level 3 severity indicator. Overall, the VIF values for the clinical and behavioral predictors were below the conservative threshold of 5.0, with the exception of the Level 3 severity indicator, which showed a slightly higher value and was therefore interpreted with caution. These findings indicate generally low to moderate collinearity among the predictors, with no evidence of substantial multicollinearity affecting the clinical and behavioral variables included in the final models.

The overall model showed good model fit, with a classification accuracy of 83.7% and an area under the ROC curve of 0.882, indicating good discriminative performance and calibration. The combined RBS-R Ritualistic/Sameness score was independently associated with TW. For every 1-point increase on this behavioral subdomain, the odds of presenting with TW increased by approximately 54.9%. Female sex was also associated with a significantly higher likelihood of presenting with TW compared to males.

Consistent with the linear regression findings, after adjusting for core repetitive behaviors, neither sleep disturbances, feeding selectivity, age, nor ASD severity levels showed independent predictive value for TW. The complete multivariable logistic model parameters, including odds ratios, Wald statistics, and confidence intervals, are reported in Table 4.

### 4.8. Sensitivity Analyses: Addressing Potential Circularity

Because the RBS-R Sameness subscale contains an item directly addressing TW (Item 32: “Walks in a certain way”), we performed a series of rigorous sensitivity analyses to exclude the possibility of construct overlap or circularity inflating our findings.

First, we examined the direct association between clinically observed TW and Item 32, which revealed a significant correlation (Mann–Whitney *U* = 10,419.0, raw *p* < 0.001, rank-biserial correlation r = 0.256), with both groups presenting a median score of 0.00, IQR [0.00–1.00]. This identical median score represents a skewed, zero-inflated distribution of this individual item, meaning that this baseline bivariate association must be interpreted with caution.

We then reconstructed all relevant RBS-R scores by completely removing Item 32 from the Sameness subscale, the combined Ritualistic/Sameness subdomain, and the RBS-R Total Score. Even after the complete exclusion of this item, the differences between the TW and non-TW groups remained significant across all reconstructed metrics. Specifically, the reconstructed Sameness score excluding Item 32 showed a median of 7.00, IQR [6.00–8.50] in the TW group compared to 4.00, IQR [2.00–5.00] in the non-TW group (*U* = 14,067.5, *p* < 0.001, rank-biserial correlation r = 0.696). The reconstructed Ritualistic/Sameness score without Item 32 was also significantly elevated in TW children (median: 12.00, IQR [9.00–13.00]) compared to the non-TW group (median: 7.00, IQR [5.00–9.00]; *U* = 14,002.0, *p* < 0.001, rank-biserial correlation r = 0.688). Similarly, the reconstructed RBS-R Total Score without Item 32 showed a robust difference between groups (median: 25.00, IQR [22.00–28.00] vs. median: 16.00, IQR [12.00–19.00]; *U* = 15,408.5, *p* < 0.001, rank-biserial correlation r = 0.858).

Second, we re-estimated the multivariable binary logistic regression model for the presence of TW using the reconstructed Ritualistic/Sameness score that completely excluded Item 32. The sensitivity model showed very similar fit, calibration, and discriminative power compared to the primary model, exhibiting a −2 log-likelihood of 216.413, a Cox & Snell R^2^ of 0.346, a Nagelkerke pseudo-R^2^ of 0.501, and an area under the ROC curve of 0.875 (Hosmer–Lemeshow goodness-of-fit test *p* = 0.448). The reconstructed Ritualistic/Sameness score remained a statistically significant variable independently associated with TW. For every 1-point increase on this reconstructed subdomain, the odds of presenting with TW increased by 53.5% (B = 0.429, SE = 0.065, Wald = 43.33, Odds Ratio = 1.535, 95% confidence interval [1.351, 1.745], *p* < 0.001). These sensitivity analyses provide additional support for the observed association between TW and behavioral rigidity, suggesting that the finding is not entirely explained by potential psychometric overlap or construct circularity.

**Figure 5:**
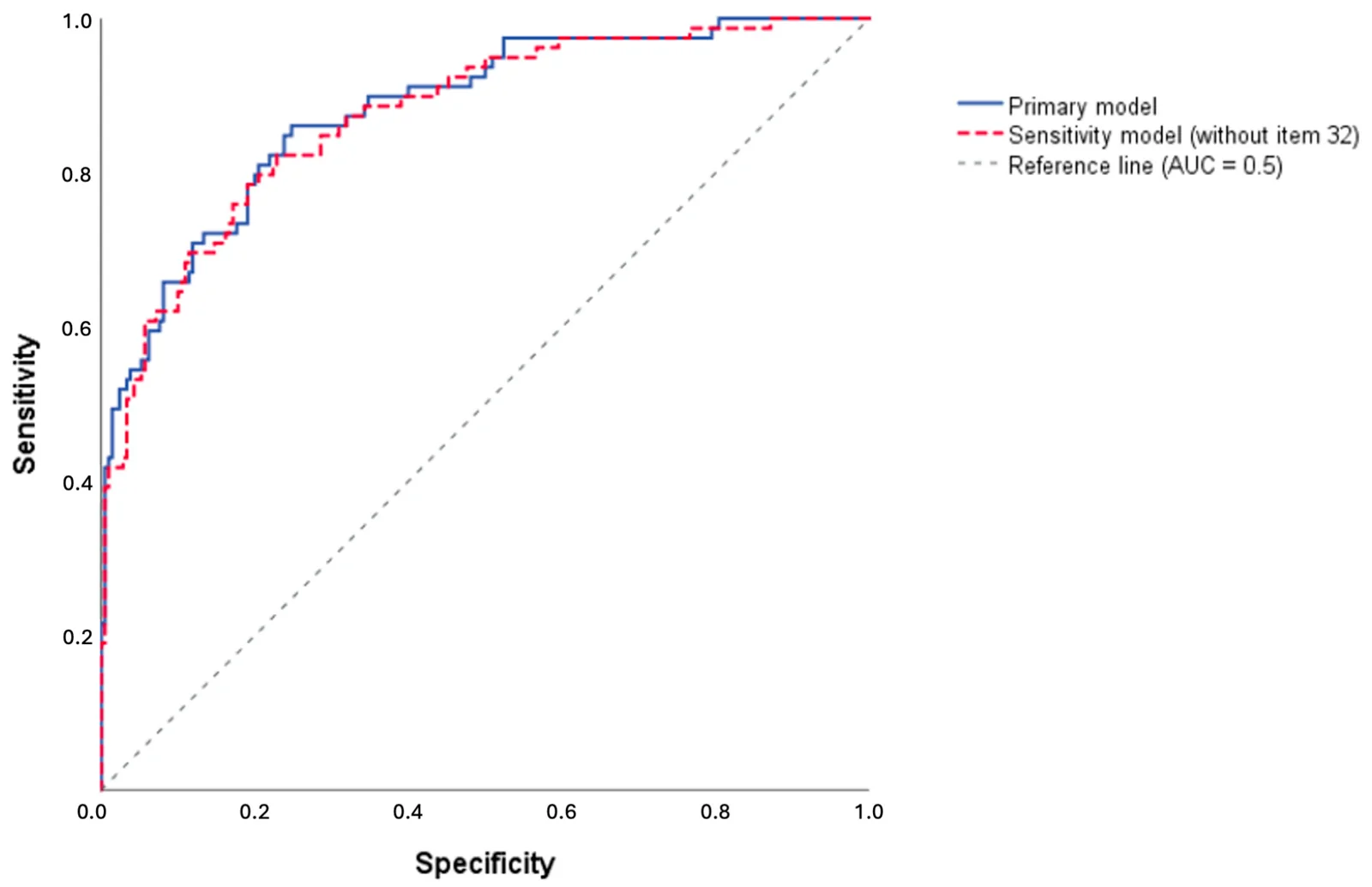
ROC curves comparing the primary and sensitivity logistic regression models for TW in children with ASD. The primary model (solid blue line; apparent AUC = 0.882, 95% CI: 0.832–0.932, *p* < 0.001) includes the full RBS-R Ritualistic/Sameness score along with SDSC total score, BAMBI score, chronological age, sex, and ASD severity level as covariates. The sensitivity model (dashed red line; apparent AUC = 0.875, 95% CI: 0.824–0.926, *p* < 0.001) replaces the full score with the reconstructed RBS-R Ritualistic/Sameness score excluding Item 32 to eliminate potential construct overlap and circularity. The diagonal grey dashed line represents the reference chance line (AUC = 0.5). Legend: ASD = autism spectrum disorder; AUC = area under the ROC curve; BAMBI = Brief Autism Mealtime Behavior Inventory; CI = confidence interval; SDSC = Sleep Disturbance Scale for Children; RBS-R = Repetitive Behavior Scale-Revised; ROC = Receiver Operating Characteristic; TW = toe walking.

The visual comparison of the receiver operating characteristic (ROC) trajectories of both the primary and sensitivity models is presented in Figure 5.

## 5. Discussion

The present study provides a multidimensional characterization of TW in children with ASD, integrating motor, behavioral, and sleep-related domains within a clinically meaningful framework.

Our findings extend previous observations by demonstrating that TW was associated with specific domains of repetitive behavior, particularly ritualistic/sameness patterns, after adjustment for the variables included in the multivariable model. Although children with TW also showed higher scores on several clinical and behavioral measures in the unadjusted group comparisons, the persistence of the association with the selected repetitive-behavior domain after adjustment suggests that this relationship cannot be fully accounted for by the covariates included in the model. This distinction is clinically relevant, as it allows us to hypothesize that TW may represent a motor feature associated with specific aspects of behavioral organization in addition to its association with broader clinical severity. Several neurodevelopmental models have proposed that executive-function and sensory-processing differences may contribute to motor behavior in ASD [32,33,34]. Although these frameworks provide a useful context for interpreting the present findings, executive functioning and sensory processing were not directly evaluated in this study. Consequently, the observed associations should be interpreted as phenotypic rather than mechanistic and specific repetitive-behavior domains should not be interpreted as evidence of underlying executive or sensory-processing mechanisms.

From this perspective, TW may be conceptualized as a motor feature that may co-occur with repetitive behavioral domains, rather than an isolated gait anomaly. The observed relationship with ritualistic and sameness behaviors is consistent with the association of motor and behavioral features in ASD, although the specific mechanisms underlying this association remain uncertain. The observed relationship with ritualistic and sameness behaviors may reflect the broader clinical co-occurrence of motor and behavioral features related to behavioral predictability and rigidity [19], although executive control and environmental structuring were not directly assessed in the present study. Preclinical and neurobiological studies have proposed several mechanisms that may contribute to motor abnormalities in ASD, including alterations in brain connectivity, synaptic plasticity, cerebello-striatal circuitry, and glutamatergic, GABAergic, and dopaminergic signaling [32,35,36,37,38]. These findings provide a theoretical context for the observed motor-behavioral associations, but none of these mechanisms were directly evaluated in the present study.

The observed correlation between TW and the total RBS-R score should be interpreted within the context of the descriptive comparison between the two clinical groups, as the total score includes the behavioral domains subsequently examined individually. Accordingly, the multivariable models provide the most informative evidence regarding the adjusted association between TW and specific repetitive-behavior domains.

In this cohort, repetitive behavior scores and sleep-disturbance scores were positively associated (Spearman’s ρ = 0.532, 95% CI: 0.443–0.610, *p* < 0.001), supporting previous evidence of a relationship between sleep disturbances and behavioral phenotypes in children with ASD. While sleep disturbances, particularly those related to sleep continuity and stability, may clinically co-occur with behavioral rigidity and repetitive patterns [24], the cross-sectional design of this study and the reliance on a shared caregiver informant limit any conclusions about directionality, causal pathways, or common regulatory mechanisms.

In the present multivariable model, the association between TW and the selected repetitive-behavior domain remained statistically significant after adjustment for sleep, feeding, age, sex, and ASD severity, whereas the coefficients for sleep and feeding were not statistically significant. This statistical attenuation does not demonstrate a biological dissociation or prove the absence of a relationship; rather, it reflects how shared clinical variance is accounted for once core repetitive behaviors are controlled. A key aspect of the present findings is difference between the unadjusted associations observed across several clinical domains and the persistence of the association with the selected repetitive-behavior domain after multivariable adjustment. While TW was strongly associated with specific RBS-R subscales, it was not independently predicted by sleep disturbances or feeding behavior when behavioral variables were included in multivariate models. This finding suggests that, within the variables included in the present model, the association between TW and the selected repetitive-behavior domain was not fully explained by sleep, feeding, age, sex, or ASD severity. It does not, however, exclude residual confounding or establish a preferential biological relationship between motor and repetitive behaviors. Such a pattern indicates that TW may be associated with the clinical organization of repetitive behaviors rather than solely representing a nonspecific manifestation of global impairment [9]. More broadly, motor-control and postural-control differences are well-documented features of the ASD phenotype, with atypical mediolateral postural sway during standing having been shown to correlate directly with the severity of restricted and repetitive behaviors [39]. Crucially, the relationship between motor atypicalities and core autism symptoms is not limited to concurrent motor control but extends back to early developmental trajectories. Longitudinal and cross-sectional evidence indicates that later age of attainment of early motor milestones, such as sitting and standing, significantly predicts the concurrent severity of restricted and repetitive behaviors [32]. Delays in these early milestones, particularly independent walking onset, also carry broader developmental significance, showing strong associations with poorer cognitive and motor performance quotients on standardized developmental scales in preschool-aged children with ASD [40]. At a translational level, these motor-behavioral overlaps are hypothesized to involve subcortical neurobiological systems, particularly basal-ganglia pathways, which play a critical role in both motor execution and the gating of repetitive behaviors [41]. These converging findings support the relevance of motor-behavioral relationships in ASD, while their underlying neurobiological substrates remain to be established.

From a clinical standpoint, this interpretation has important implications. TW, often regarded as a peripheral or nonspecific sign, may instead provide relevant information about the underlying behavioral profile of the child. Nevertheless, these findings suggest a potential hypothesis for clinical practice rather than a validated diagnostic recommendation. Our data do not imply that TW can be utilized to identify a specific behavioral phenotype with sufficient accuracy for direct clinical decision-making. Instead, it is reasonable to suggest that clinicians who observe persistent TW in children with ASD may consider this motor feature as an additional clinical feature that may prompt a more detailed, multidimensional assessment. Specifically, its clinical presentation may warrant further evaluation of repetitive behaviors as well as sensory processing, sleep disturbances, and overall functional daily impact.

The inclusion of the RBS-R Global Rating Score [30], which reflects caregiver perception of daily interference, further emphasizes the importance of subjective burden. This perspective underscores the importance of considering not only the frequency or intensity of repetitive behaviors but also their perceived functional impact. This aligns with a patient-centered approach and may help guide clinical prioritization and intervention strategies.

Taken together, these findings support the interpretation of TW as part of a multidimensional clinical profile, in which motor, behavioral, and sleep domains may co-occur without being fully redundant [9]. This perspective is consistent with conceptual frameworks that view ASD as a dynamic disorder of developmental regulation [42], where co-occurring alterations in arousal, behavior, and motor output are hypothesized to reflect different clinical expressions of underlying regulatory processes.

In this context, frameworks aimed at phenotypic stratification—such as those integrating sleep, behavioral, and physiological domains—may provide a useful structure for future research. Similarly, increasing attention has been directed toward biological correlates of regulatory dysfunction, including the complex relationships between sleep disturbances, neurobiological alterations, core ASD symptoms, and psychiatric comorbidities [43]. In a highly exploratory context, pre-clinical and clinical investigations have proposed potential biological correlates of these co-occurring features, such as sleep dysregulation being associated with altered neuroendocrine pathways, including leptin levels [44]. These findings remain preliminary and provide context for future research rather than direct evidence for the mechanisms underlying the present associations.

Importantly, previous work on this cohort has demonstrated that TW is associated with sleep disturbances and gastrointestinal symptoms, suggesting a broader multisystem involvement [9]. The present study builds upon these findings by identifying a more specific behavioral link for this motor phenomenon, thereby refining its clinical interpretation within the ASD phenotype. These findings also have relevance for conceptual discussions concerning the remarkable clinical heterogeneity of ASD and the ongoing search for distinct, homogeneous endophenotypes [45]. Within this framework, TW may represent a clinically relevant motor feature potentially associated with altered sensory integration, repetitive motor entrainment mechanisms, and broader neurodevelopmental features involving behavioral rigidity and sleep instability.

Consistent with emerging person-centered models of ASD heterogeneity [46], recent advances in psychiatric genetics have attempted to decompose clinical and phenotypic heterogeneity into distinct, underlying genetic programs [47]. Although these genetic models do not directly evaluate motor features such as TW, they provide a valuable conceptual framework for understanding the biological heterogeneity underlying clinical phenotypes. However, the present study did not assess genetic or molecular variables, and whether TW is enriched in any biologically defined subgroup remains unknown. Future research may therefore investigate whether TW and specific patterns of repetitive behavior are differentially represented across clinically or biologically defined subgroups of ASD.

These findings also raise the hypothesis that interventions addressing repetitive behaviors could be explored in future studies to determine whether changes in behavioral organization are accompanied by changes in motor manifestations such as TW. Moreover, from a rehabilitative perspective, atypical motor features in pediatric ASD, such as TW, represent a significant clinical challenge, as no established clinical gold standard currently exists for their management. Systematic reviews addressing both ASD-specific cohorts and idiopathic TW consistently indicate a very low certainty of evidence for both conservative and surgical interventions [48,49,50]. In children with ASD, TW may represent a complex motor feature involving sensory, behavioral, and motor-regulatory factors rather than an isolated musculoskeletal abnormality [9,50]. Accordingly, although orthopedic procedures such as Achilles tendon lengthening may provide structural correction, their effectiveness in autistic children may be limited by the risk of behavioral recurrence [50]. Unimodal conservative approaches, including isolated physical therapy or orthotic management, have yielded variable outcomes [48,50,51]. Multimodal interventions, combining botulinum toxin, serial casting, and orthotic stabilization [52], as well as structured behavioral and motor-based programs, have shown more favorable results [53]. However, the available evidence remains limited, with much of the literature on behavioral and rehabilitative interventions deriving from small pilot studies, case reports, or single-case experimental designs, thereby restricting the generalizability of these findings [50,53]. Recent evidence has also highlighted the potential value of integrated rehabilitative approaches, including neuropsychomotor therapy (TNPEE) and animal-assisted interventions, in supporting developmental and motor outcomes in children with neurodevelopmental disorders [42,54,55]. Other preliminary reports have explored biomechanically oriented strategies, such as backward walking exercises, as potential approaches to gait retraining [56]. Although these findings are encouraging, the available evidence remains heterogeneous and largely based on small or case-based studies [50,56].

## 6. Limitations

Several limitations should be considered when interpreting these findings.

First, the cross-sectional design of the study does not allow for causal inferences. The observed associations between TW, repetitive behaviors, and sleep disturbances reflect concurrent relationships and do not clarify temporal or mechanistic pathways.

Second, the assessment of key variables relied on parent-reported questionnaires, including the RBS-R, SDSC, and BAMBI. While these instruments are widely validated and clinically informative, they are inherently subject to reporting bias and may reflect parental perception rather than objective behavioral quantification. Moreover, because the SDSC, BAMBI, and RBS-R were completed by the same primary caregiver, associations among these behavioral measures may have been partially influenced by common-method (reporter) bias.

Third, although the use of item-level RBS-R data represents a methodological strength, the absence of direct observational or instrument-based measures of motor activity and sleep (e.g., actigraphy or polysomnography) means that any discussion of potential physiological mechanisms remains hypothetical and should be interpreted as a conceptual framework rather than as evidence derived from the present study. Moreover, the lack of independent statistical significance for sleep, feeding, age, and autism severity in our multivariable models should not be interpreted as evidence of a true absence of relationship; rather, this non-significance may reflect limitations in statistical power, residual confounding, measurement error, or model specification in our sample.

Fourth, the study did not include biological or neurophysiological markers. As a result, the proposed interpretation of a shared regulatory substrate remains conceptual and cannot be directly supported by objective measures of neural or metabolic function.

The assessment of TW has some limitations. Although TW was identified through direct clinical observation during spontaneous barefoot walking by experienced child neuropsychiatrists and confirmed within a multidisciplinary clinical assessment including physiatric and orthopedic evaluation whenever cooperation with the examination was possible, the parent observational study was not specifically designed as an instrumented gait-analysis study. Consequently, detailed quantitative gait characteristics—such as the proportion of TW steps, cadence, walking speed, standardized walking distance, repeated trials, or instrument-based gait measurements—were neither collected nor retained in the analytical dataset. Likewise, formal inter-rater reliability was not evaluated. Therefore, the present findings should be interpreted as referring to a clinically observable motor phenotype identified during routine multidisciplinary assessment rather than as a quantitative biomechanical characterization of gait. Additionally, because in the present cohort the classification of TW was based strictly on direct clinical observation during the evaluation sessions, we could not account for intermittent or context-specific TW that might occur exclusively in other settings, such as the home environment. The binary classification of TW may also have limited the ability to capture variability in frequency, severity, or clinical presentation. The potential conceptual overlap between TW and RBS-R Item 32 was specifically addressed through a sensitivity analysis, which yielded consistent results, although the possibility of some construct overlap cannot be completely excluded.

In addition, because the present work is a secondary analysis of an anonymized multicenter dataset, center identifiers were not retained in the analytical database. Consequently, potential center-related variability could not be formally assessed or incorporated into the statistical models. Our multivariable models did not account for several potentially confounding clinical factors, such as standardized cognitive level, speech development, sensory hypersensitivity, anxiety, or rehabilitative interventions. Although cognitive screening was routinely performed during the initial clinical intake to confirm the diagnosis, continuous cognitive scores were not available in this anonymized secondary dataset. Consequently, we cannot completely rule out the influence of these unadjusted variables, which limits our ability to interpret the observed motor-behavioral associations as strictly specific to repetitive behaviors. Future prospective studies should also incorporate standardized and detailed tracking of rehabilitative exposure to determine whether specific habilitative pathways influence the clinical expression, persistence, and response to treatment of TW in children with ASD. Furthermore, the multicenter sample was recruited from tertiary academic centers, which may introduce referral and selection bias and may limit the generalizability of the findings to community-based populations, children with milder ASD, or children who do not receive specialized neuropsychiatric care.

Since TW was classified independently through direct clinical observation rather than caregiver report, the possibility that caregiver-related reporting tendencies contributed to the observed associations among questionnaire-derived variables cannot be completely excluded. Additionally, we must acknowledge that several individual subscales of the caregiver-reported RBS-R exhibited low Cronbach’s α values in our sample. While this represents a psychometric limitation, it reflects the clinical reality of assessing highly heterogeneous and fluctuating repetitive phenotypes in preschool-aged children, where individual topographies do not cluster as consistently as they do in older cohorts. Moreover, the classification accuracy (83.7%) and AUC (0.882) of our model represent apparent, optimistic performance metrics calculated on the same derivation sample. Because our dataset lacks internal or external validation on an independent cohort, these metrics must be interpreted as purely descriptive of our sample rather than generalizable predictive performance. The relatively complex multivariable model in relation to the available sample size also raises the possibility of model overfitting, particularly given the number of predictors included and the absence of independent validation. Accordingly, the reported model performance should not be interpreted as demonstrating generalizable predictive accuracy.

Although the parent cohort followed a prospective observational design, registration on ClinicalTrials.gov occurred after study initiation. Therefore, interpret this secondary, hypothesis-driven analysis in the context of a retrospectively registered observational cohort.

Finally, the analysis was restricted to children with ASD, and typically developing controls were not included in the present phase. While this choice was methodologically appropriate for phenotypic characterization, it limits our ability to determine whether the observed association between TW and repetitive behaviors is specific to ASD or reflects a broader relationship in other developmental conditions. To address this clinical specificity, future studies should include typically developing children alongside other clinical comparison groups—including children with developmental language disorder, intellectual disability, sensory processing disorder, cerebral palsy, and idiopathic TW—depending on the research question.

## 7. Conclusions

In conclusion, the findings of this cross-sectional study indicate that TW is significantly associated with higher scores in specific restricted and repetitive behavior domains—particularly stereotyped and ritualistic/sameness behaviors—within this pediatric ASD cohort. These findings are exploratory and should not be interpreted as evidence of absolute specificity, causality, or a shared neurobiological mechanism. Persistent TW may provide clinically relevant information warranting further, more detailed assessment of a child’s broader behavioral profile. However, this clinical suggestion must be treated strictly as a hypothesis requiring future prospective and longitudinal validation.

## Figures and Tables

**Table 1:** Demographic and clinical baseline comparisons between ASD children with and without TW.

Variable	TW (*N* = 79)	Non-TW (*N* = 210)	Test Statistic (*U* or χ^2^)	*p*-Value; *p*_FDR_	Effect Size (r or *V*)
Age (years)	5.94 ± 0.77; 5.90 [5.40–6.50]	5.91 ± 0.78; 5.90 [5.40–6.50]	*U* = 8443.5	0.811; 0.811	r = 0.018
Sex (F/M)	F: 34 (43%) M: 45 (57%)	F: 39 (18.6%) M: 171 (81.4%)	χ^2^ = 16.93	<0.001; <0.001	*V* = 0.242
ADOS-2 Total score	16.70 ± 3.92; 18.00 [13.00–20.00]	13.68 ± 3.68; 13.00 [11.00–15.00]	*U* = 11,655.0	<0.001; <0.001	r = 0.405
ADI-R score	28.62 ± 5.50; 30.00 [27.00–32.00]	25.27 ± 5.75; 27.00 [20.00–29.00]	*U* = 11,015.5	<0.001; <0.001	r = 0.328
SDSC Total score	74.70 ± 8.12; 76.00 [69.50–80.50]	68.28 ± 7.97; 68.00 [62.00–73.00]	*U* = 11,830.5	<0.001; <0.001	r = 0.426
BAMBI score	73.27 ± 13.23; 80.00 [60.00–86.00]	68.00 ± 12.56; 62.00 [58.00–83.75]	*U* = 10,074.0	0.005; 0.005	r = 0.214
ASD Level (1/2/3)	L1: 13 (16.5%) L2: 16 (20.3%) L3: 50 (63.3%)	L1: 82 (39%) L2: 79 (37.6%) L3: 49 (23.3%)	χ^2^ = 40.94	<0.001; <0.001	*V* = 0.376

Note: continuous variables are reported as Mean ± SD and Medians [IQR]; categorical variables are reported as counts and percentages. Rank-biserial correlation coefficients are reported as absolute values (positive terms) to facilitate intuitive interpretation; a positive value consistently indicates higher clinical or behavioral scores in the TW group compared to the non-TW group. Legend: ADI-R = Autism Diagnostic Interview-Revised; ADOS-2 = Autism Diagnostic Observation Schedule—Second edition; BAMBI = Brief Autism Mealtime Behavior Inventory; F = female; M = male; SD = standard deviation; SDSC = Sleep Disturbance Scale for Children; TW = toe walking.

**Table 2:** Bivariate Spearman correlation matrix across motor, behavioral, sleep, and clinical severity variables.

Association	Correlation Coefficient (ρ)	*p*-Value	Clinical Interpretation
TW × Stereotyped behaviors	0.588	<0.001	Moderate-to-strong positive association
TW × Ritualistic/sameness	0.549	<0.001	Moderate positive association
TW × RBS-R Total Score	0.670	<0.001	Strong positive association
SDSC Total score × RBS-R Total Score	0.532	<0.001	Moderate positive association
ASD Level × RBS-R Total Score	0.535	<0.001	Moderate positive association
Age × RBS-R Total Score	−0.058	0.325	No statistically significant association

Note: TW is coded as 0 = absent, 1 = present; ASD severity level is treated as an ordinal factor; *p*-values are reported as raw. Legend: ASD = Autism Spectrum Disorder; RBS-R = Repetitive Behavior Scale-Revised; SDSC = Sleep Disturbance Scale for Children; TW = toe walking.

**Table 3:** Multivariable linear regression model associated with RBS-R Total Score.

Covariate	B	SE	95% CI of B	Beta	t	*p*-Value	Model
Intercept	5.309	4.223	[−3.005, 13.623]	-	1.257	0.210	F(7, 281) = 61.77; *p* < 0.001 *; R^2^ = 0.606; Adj. R^2^ = 0.596
TW	7.771	0.593	[6.603, 8.939]	0.551	13.098	<0.001 *	
BAMBI score	0.081	0.021	[0.039, 0.123]	0.167	3.838	<0.001 *	
ASD Level 2	1.858	0.701	[0.478, 3.237]	0.139	2.651	0.008 *	
ASD Level 3	2.785	1.153	[0.516, 5.053]	0.210	2.416	0.016 *	
SDSC Total Score	0.100	0.054	[−0.007, 0.206]	0.134	1.836	0.067	
Age	−0.510	0.304	[−1.108, 0.087]	−0.063	−1.680	0.094	
Sex (Male dummy)	−0.116	0.562	[−1.224, 0.992]	−0.008	−0.206	0.837	

Note: Asterisk (*) denotes significant results. Legend: ASD = Autism Spectrum Disorder; BAMBI = Brief Autism Mealtime Behavior Inventory; SDSC = Sleep Disturbance Scale for Children; RBS-R = Repetitive Behavior Scale-Revised; TW = toe walking.

**Table 4:** Multivariable binary logistic regression model associated with the presence of TW.

Covariate	B	SE	Wald	OR	95% CI of OR	*p*-Value	Model
Intercept	−4.992	3.146	2.517	0.007	-	0.113	−2 Log-Likelihood = 210.10; Cox & Snell R^2^ = 0.36; Nagelkerke R^2^ = 0.521; McFadden R^2^ = 0.380. Overall Classification Accuracy = 83.7%; AUC = 0.882; Hosmer–Lemeshow goodness-of-fit test: χ^2^ = 13.505 (*p* = 0.096)
RBS-R Ritualistic/Sameness	0.483	0.064	46.512	1.549	[1.366, 1.757]	<0.001 *	
Sex (Male dummy)	−1.533	0.390	15.425	0.216	[0.101, 0.464]	<0.001 *	
SDSC Total Score	0.018	0.040	0.210	1.019	[0.941, 1.102]	0.647	
BAMBI score	−0.020	0.016	1.505	0.980	[0.949, 1.012]	0.220	
Age	0.153	0.230	0.440	1.165	[0.741, 1.830]	0.507	
ASD Level 2	−0.370	0.561	0.436	0.691	[0.230, 2.072]	0.509	
ASD Level 3	0.695	0.833	0.696	2.004	[0.391, 10.261]	0.404	

Note: Asterisk (*) denotes significant results. Legend: ASD = Autism Spectrum Disorder; AUC = Area Under the ROC Curve; BAMBI = Brief Autism Mealtime Behavior Inventory; OR = Odds Ratio; SDSC = Sleep Disturbance Scale for Children; RBS-R = Repetitive Behavior Scale-Revised; TW = toe walking.

## Data Availability

The data presented in this study are available on reasonable request from the corresponding author. Data are not publicly available due to privacy and ethical restrictions related to pediatric clinical data.

## Supplementary Materials

The following supporting information can be downloaded at: https://www.mdpi.com/article/10.3390/medicina62091764/s1, Table S1. Item-level RBS-R comparisons between ASD children with and without TW.

## References

[1] American Psychiatric Association (2022). Diagnostic and Statistical Manual of Mental Disorders (DSM-5-TR).

[2] Micai M., Fatta L.M., Gila L., Caruso A., Salvitti T., Fulceri F., Ciaramella A., D’AMico R., Del Giovane C., Bertelli M. (2023). Prevalence of co-occurring conditions in children and adults with autism spectrum disorder: A systematic review and meta-analysis. Neurosci. Biobehav. Rev..

[3] Mirizzi P., Esposito M., Ricciardi O., Bove D., Fadda R., Caffò A.O., Mazza M., Valenti M. (2025). Food Selectivity in Children with Autism Spectrum Disorder and in Typically Developing Peers: Sensory Processing, Parental Practices, and Gastrointestinal Symptoms. Nutrients.

[4] Engström P., Tedroff K. (2018). Idiopathic Toe-Walking: Prevalence and Natural History from Birth to Ten Years of Age. J. Bone Jt. Surg. Am..

[5] Leyden J., Fung L., Frick S. (2019). Autism and toe-walking: Are they related? Trends and treatment patterns between 2005 and 2016. J. Child. Orthop..

[6] Valagussa G., Purpura G., Balatti V., Trentin L., Signori A., Grossi E. (2024). Quantitative assessment of tip-toe behavior in individuals with autism spectrum disorder and intellectual disability: A cross-sectional study. Autism Res..

[7] Donne J.H., Pacey V., Polt D., Powell J.A., Fahey M.C., Williams C.M. (2026). Investigating the Relationship Between Sensory Processing, Pain and Toe Walking Gait: A Survey Study. J. Paediatr. Child Health.

[8] Perin C., Valagussa G., Mazzucchelli M., Gariboldi V., Cerri C.G., Meroni R., Grossi E., Cornaggia C.M., Menant J., Piscitelli D. (2020). Physiological Profile Assessment of Posture in Children and Adolescents with Autism Spectrum Disorder and Typically Developing Peers. Brain Sci..

[9] Costanza C., Gallai B., Sorrentino M., Gnazzo M., Pisanò G., Parisi L., Germanò E., Maltese A., Esposito M., Roccella M. (2025). The Prevalence and Clinical Significance of Toe Walking in Autism Spectrum Disorder: A Cross-Sectional Study in an Italian Pediatric Sample. Medicina.

[10] de Angeli L.R.A., Serafim B.L.C., Masquijo J.J. (2025). The Autistic Toe Walking: A Narrative Review for Interventions and Comparison with Idiopathic Toe Walking. Children.

[11] Chapek M., Kessler J. (2025). The Prevalence of Persistent Toe Walking in Children With and Without Autism Spectrum Disorder and the Odds of Subsequent Surgery. J. Foot Ankle Surg..

[12] Barrow W.J., Jaworski M., Accardo P.J. (2011). Persistent toe walking in autism. J. Child Neurol..

[13] Purpura G., Cerroni F., Carotenuto M., Nacinovich R., Tagliabue L. (2022). Behavioural Differences in Sensorimotor Profiles: A Comparison of Preschool-Aged Children with Sensory Processing Disorder and Autism Spectrum Disorders. Children.

[14] Valagussa G., Purpura G., Nale A., Pirovano R., Mazzucchelli M., Grossi E., Perin C. (2022). Sensory Profile of Children and Adolescents with Autism Spectrum Disorder and Tip-Toe Behavior: Results of an Observational Pilot Study. Children.

[15] Esposito G., Venuti P., Apicella F., Muratori F. (2011). Analysis of unsupported gait in toddlers with autism. Brain Dev..

[16] Williams C.M., Tinley P., Curtin M., Wakefield S., Nielsen S. (2014). Is idiopathic toe walking really idiopathic? The motor skills and sensory processing abilities associated with idiopathic toe walking gait. J. Child Neurol..

[17] Comparan-Meza M., Vargas de la Cruz I., Jauregui-Huerta F., Gonzalez-Castañeda R.E., Gonzalez-Perez O., Galvez-Contreras A.Y. (2021). Biopsychological correlates of repetitive and restricted behaviors in autism spectrum disorders. Brain Behav..

[18] Ferguson E.F., Spackman E., Cai R.Y., Hardan A.Y., Uljarević M. (2024). Characterizing associations between emotion dysregulation, anxiety, and repetitive behaviors in autistic youth with intellectual disability. Autism Res..

[19] Uddin L.Q., Supekar K., Lynch C.J., Cheng K.M., Odriozola P., Barth M.E., Phillips J., Feinstein C., Abrams D.A., Menon V. (2015). Brain State Differentiation and Behavioral Inflexibility in Autism. Cereb. Cortex.

[20] Smith C., Baker D.H. (2025). Neural correlates of the deployment of spatial attention, and their modulation by repetitive movements. PLoS ONE.

[21] Bieneck V., Bletsch A., Mann C., Schäfer T., Seelemeyer H., Herøy N., Zimmermann J., Pretzsch C.M., Hattingen E., Ecker C. (2021). Longitudinal Changes in Cortical Thickness in Adolescents with Autism Spectrum Disorder and Their Association with Restricted and Repetitive Behaviors. Genes.

[22] Bruni O., Mammarella V., Breda M., Gringras P., Malow B.A., Schaer M., Schroder C.M., Weiss S.K. (2026). Sleep as a viable target for early intervention in children with autism spectrum disorder: A narrative review informed by a systematic literature search. Sleep Med. Rev..

[23] Mazurek M.O., Sohl K. (2016). Sleep and Behavioral Problems in Children with Autism Spectrum Disorder. J. Autism Dev. Disord..

[24] Sevincok D., Isik C.M., Ozbek M.M., Ozbay H.C., Ozturk M. (2026). Sleep Disturbances and Behavioural Phenotypes in Children with Autism Spectrum Disorder: A Comparative Study with Typically Developing Peers. Int. J. Dev. Neurosci..

[25] Fucà E., Passarini S., Guerrera S., Dentale F., Costanzo F., Parisi M., Casula L., Pirchio S., Menghini D., Valeri G. (2025). Sleep Mediates the Association Between Emotion Dysregulation and Repetitive Behaviors in Autistic Children. J. Autism Dev. Disord..

[26] Schreck K.A., Richdale A.L. (2020). Sleep problems, behavior, and psychopathology in autism: Inter-relationships across the lifespan. Curr. Opin. Psychol..

[27] Hollway J.A., Aman M.G. (2011). Sleep correlates of pervasive developmental disorders: A review of the literature. Res. Dev. Disabil..

[28] Bruni O., Ottaviano S., Guidetti V., Romoli M., Innocenzi M., Cortesi F., Giannotti F. (1996). The Sleep Disturbance Scale for Children (SDSC) Construction and validation of an instrument to evaluate sleep disturbances in childhood and adolescence. J. Sleep Res..

[29] Lamboglia A., Romano R., Valente D., Berardi A., Cavalli G., Giovannone F., Sogos C., Tofani M., Galeoto G. (2023). Brief Autism Mealtime Behavior Inventory (BAMBI): Italian Translation and Validation. Children.

[30] Fulceri F., Narzisi A., Apicella F., Balboni G., Baldini S., Brocchini J., Domenici I., Cerullo S., Igliozzi R., Cosenza A. (2016). Application of the Repetitive Behavior Scale-Revised--Italian version--in preschoolers with autism spectrum disorder. Res. Dev. Disabil..

[31] Lam K.S.L., Aman M.G. (2007). The Repetitive Behavior Scale-Revised: Independent validation in individuals with autism spectrum disorders. J. Autism Dev. Disord..

[32] Uljarević M., Hedley D., Alvares G.A., Varcin K.J., Whitehouse A.J.O. (2017). Relationship between early motor milestones and severity of restricted and repetitive behaviors in children and adolescents with autism spectrum disorder. Autism Res..

[33] Carotenuto M., Ruberto M., Fontana M.L., Catania A., Misuraca E., Precenzano F., Lanzara V., Messina G., Roccella M., Smirni D. (2019). Executive functioning in autism spectrum disorders: A case-control study in preschool children. Curr. Pediatr. Res..

[34] Smirni D., Smirni P., Carotenuto M., Parisi L., Quatrosi G., Roccella M. (2019). Noli Me Tangere: Social Touch, Tactile Defensiveness, and Communication in Neurodevelopmental Disorders. Brain Sci..

[35] Duan J., Zeng D., Wu T., Luo Z., Jingwen G., Tan W., Zeng Y. (2025). Neural connections and molecular mechanisms underlying motor skill deficits in genetic models of autism spectrum disorders. Prog. Neurobiol..

[36] Lepping R.J., McKinney W.S., Magnon G.C., Keedy S.K., Wang Z., Coombes S.A., Vaillancourt D.E., Sweeney J.A., Mosconi M.W. (2022). Visuomotor brain network activation and functional connectivity among individuals with autism spectrum disorder. Hum. Brain Mapp..

[37] Carper R.A., Solders S., Treiber J.M., Fishman I., Müller R.-A. (2015). Corticospinal tract anatomy and functional connectivity of primary motor cortex in autism. J. Am. Acad. Child Adolesc. Psychiatry.

[38] Jagadapillai R., Tuncali I., Nagarajan N., Barnes G., Gozal E. (2026). Cerebellar Abnormalities: A Component of Autism Pathophysiology. Medicina.

[39] Bojanek E.K., Wang Z., White S.P., Mosconi M.W. (2020). Postural control processes during standing and step initiation in autism spectrum disorder. J. Neurodev. Disord..

[40] Sousa D., Queirós J.C., Tavares T., Soares S., Vaz Matos I., Prior C., Gonzaga D. (2025). Early Neurodevelopmental Milestones in Children With Autism Spectrum Disorder: A Retrospective Observational Study. Cureus.

[41] Subramanian K., Brandenburg C., Orsati F., Soghomonian J., Hussman J.P., Blatt G.J. (2017). Basal ganglia and autism—A translational perspective. Autism Res..

[42] Gnazzo M., Bargiacchi G., Esposito M., Passerini R., Varriale E., Cerroni F., Germanò E., Maltese A., Parisi L., Roccella M. (2026). Longitudinal Effects of Neuropsychomotor Therapy on Clinical Outcomes in Autism Spectrum Disorder: An 18-Month Multicenter Rehabilitation Study. Disabilities.

[43] Mazzone L., Postorino V., Siracusano M., Riccioni A., Curatolo P. (2018). The Relationship between Sleep Problems, Neurobiological Alterations, Core Symptoms of Autism Spectrum Disorder, and Psychiatric Comorbidities. JCM.

[44] Carotenuto M., Gnazzo M., Bargiacchi G., Baldini V., Umano G.R., Messina G., Monda M., Smirni D., Plazzi G., Spruyt K. (2026). Leptin as a biomarker of sleep dysregulation in children with ASD: A drug-free cohort study. Sleep Med..

[45] Waterhouse L. (2022). Heterogeneity thwarts autism explanatory power: A proposal for endophenotypes. Front. Psychiatry.

[46] Wang M., Zhang X., Zhong L., Zeng L., Li L., Yao P. (2025). Understanding autism: Causes, diagnosis, and advancing therapies. Brain Res. Bull..

[47] Litman A., Sauerwald N., Green Snyder L., Foss-Feig J., Park C.Y., Hao Y., Dinstein I., Theesfeld C.L., Troyanskaya O.G. (2025). Decomposition of phenotypic heterogeneity in autism reveals underlying genetic programs. Nat. Genet..

[48] van Kuijk A.A.A., Kosters R., Vugts M., Geurts A.C.H. (2014). Treatment for idiopathic toe walking: A systematic review of the literature. J. Rehabil. Med..

[49] Valagussa G., Trentin L., Signori A., Grossi E. (2018). Toe Walking Assessment in Autism Spectrum Disorder Subjects: A Systematic Review. Autism Res..

[50] Perricone E., Caldaci A., Cacciaguerra G., di Nora A., Parano C., Pavone V. (2026). Comparative effectiveness of conservative and surgical interventions for toe walking in children with Autism Spectrum Disorder: A systematic review. Front. Med..

[51] Bartoletta J., Tsao E., Bouchard M. (2021). A Retrospective Analysis of Nonoperative Treatment Techniques for Idiopathic Toe Walking in Children: Outcomes and Predictors of Success. PM R.

[52] Manfredi F., Riefoli F., Coviello M., Dibello D. (2022). The Management of Toe Walking in Children with Autism Spectrum Disorder: “Cast and Go”. Children.

[53] Semino M., Riccio E., Giannatiempo S., Cavallini F., Vascelli L. (2025). Evaluating a Treatment Package to Reduce Toe Walking and Improve Ankle Mobility in Children with Autism Spectrum Disorder: A Multi-Component Intervention. Behav. Anal. Pract..

[54] Gnazzo M., Bargiacchi G., Baldini V., Esposito M., Passerini R., Varriale E., Cerroni F., Germanò E., Maltese A., Parisi L. (2026). Chronological Age and Adaptive Outcomes Following Neuropsychomotor and Aquatic Interventions in Children with Autism: A Secondary Analysis. Children.

[55] Gnazzo M., Pisanò G., Hedili O., Baldini V., Cesaroni C.A., Esposito A., D’ONofrio T., Terracciano A.M., Bargiacchi G., Carotenuto M. (2026). Dog-assisted therapy and motor coordination in children with autism spectrum disorder: An exploratory pre-post study. Child Neuropsychol..

[56] Akbuga E. (2026). Preliminary evidence for backward walking to reduce toe walking in autism spectrum disorder: A single-case experimental pilot study. Acta Psychol..

